# Point of care ultrasound training for internal medicine: a Canadian multi-centre learner needs assessment study

**DOI:** 10.1186/s12909-018-1326-8

**Published:** 2018-09-20

**Authors:** Kathryn Watson, Ada Lam, Shane Arishenkoff, Samantha Halman, Neil E. Gibson, Jeffrey Yu, Kathryn Myers, Marcy Mintz, Irene W. Y. Ma

**Affiliations:** 1 Department of Medicine, University of Calgary, Calgary, AB Canada; 2 Department of Medicine, University of Alberta, Edmonton, AB Canada; 3 Department of Medicine, University of British Columbia, Vancouver, BC Canada; 4 Department of Medicine, University of Ottawa, Ottawa, ON Canada; 5 Department of Medicine, Western University, London, ON Canada; 6 W21C, University of Calgary, 3330 Hospital Dr NW, Calgary, AB T2N 4N1 Canada

**Keywords:** Point-of-care ultrasound, Education needs assessment, Curriculum development, Internal medicine

## Abstract

**Background:**

Significant gaps currently exist in the Canadian internal medicine point-of-care ultrasound (POCUS) curriculum. From a learner’s perspective, it remains unknown what key POCUS skills should be prioritized. This needs assessment study seeks to establish educational priorities for POCUS for internal medicine residents at five Canadian residency training programs.

**Methods:**

All internal medicine trainees [postgraduate year (PGY) 1–5] from five internal medicine residency training programs in Canada (*n* = 598) were invited to complete an online survey on 15 diagnostic POCUS applications, 9 bedside procedures, and 18 POCUS knowledge items. For POCUS applications and procedures, participants were asked how applicable they are to patient care in internal medicine and the participants’ reported skills in those domains. Self-reported knowledge and skills were rated on a 5-point Likert scale, where 1 = very poor and 5 = very good. Applicability was rated, where 1 = not at all applicable and 5 = very applicable.

**Results:**

A total of 253 of 598 residents (42%) participated in our study. Data from one centre (*n* = 15) was removed because of low response rate (15%) and significant baseline differences between those trainees and the remaining participants. Of the remaining analyzable data from four training programs (*n* = 238), participants reported highest applicability to internal medicine for the following applications and procedures: *identifying ascites/free fluid* [mean applicability score of 4.9 ± standard deviation (SD) 0.4]; *gross left ventricular function* (mean 4.8 ± SD 0.5) and *pericardial effusion* (mean 4.7 ± SD 0.5); *thoracentesis* (mean score 4.9 ± SD 0.3), *central line insertion* (mean 4.9 ± SD 0.3), and *paracentesis* (mean 4.9 ± SD 0.3), respectively. Overall reported knowledge/skills was low, with skill gaps being the highest for *identifying deep vein thrombosis* (mean gap 2.7 ± SD 1.1), *right ventricular strain* (mean 2.7 ± SD 1.1), and *gross left ventricular function* (mean 2.7 ± SD 1.0).

**Conclusions:**

Many POCUS applications and procedures were felt to be applicable to the practice of internal medicine. Significant skill gaps exist in the four Canadian training programs included in the study. POCUS curriculum development efforts should target training based on these perceived skill gaps.

**Electronic supplementary material:**

The online version of this article (10.1186/s12909-018-1326-8) contains supplementary material, which is available to authorized users.

## Background

There is an increasing recognition of the value of point-of-care ultrasound (POCUS) in the practice of internal medicine. Its use in the guidance of procedures, such as central venous catheterization and thoracentesis, has been shown to improve patient safety [1, 2] and is considered to be the standard of care [2–9]. Additionally, the use of ultrasound as an adjunct to the physical examination to clarify clinical findings [10–12] and its ability to identify important clinical conditions at the bedside in the acutely ill patient is also increasingly recognized [13].

Appropriate training is integral for incorporating POCUS into the practice of internal medicine. POCUS involves a complex set of skills, including image acquisition, image interpretation, and integration of findings that require consideration of the patient’s clinical context and pre-test probability [14, 15]. To practice POCUS safely, the trainee must have sufficient medical and sonographic knowledge of possible differential diagnoses [16], as well as an awareness and insight into the limitations of both POCUS use in general and one’s own skill limitations. Given this complexity, it is not surprising the performance of POCUS is highly operator dependent and that the learning curves differ significantly depending on the application and the learner in question [17–19]. In the hands of untrained or inadequately trained POCUS operators, medical errors may pose significant safety concerns for the patient [20]. A growing body of evidence indicates that dedicated training is indeed necessary to attain proficiency in POCUS [5, 18, 21] and that training may mitigate against potential harm from common POCUS pitfalls [22, 23]. Targeting skills and knowledge gaps is therefore critical to POCUS education.

For curriculum development, performing a needs assessment is one of the key recommended first steps [24]. From a curriculum development standpoint, consensus was recently established regarding what POCUS elements should be included in a Canadian internal medicine training curriculum [25, 26]. However, this work stemmed from the viewpoint of the educator. Little is known from the trainees’ perspective, both in terms of what they feel are needed skills for internal medicine and what skill gaps exist. For adult learners, recognition of their perspectives and needs is critical for curriculum success [24, 27].

Literature to date suggests gaps exist in POCUS training. One study involving learners at the University of Illinois at Chicago and Northwestern University shows that learners felt generally incompetent in the use of ultrasound [28].These results are not surprising, considering that, based on results from a survey administered to internal medicine Program Directors in 2012, only 25% reported having a formal POCUS curriculum [29]. Significant training gaps are similarly present in Canada [30]. Specifically, while 100% of the internal medicine Canadian residency and fellowship program directors believed that POCUS should be taught and used, only 53% had actually integrated it into their residency-training programs [30].

The aim of this study is to establish the current reported skill levels and perceived POCUS needs of internal medicine trainees at five Canadian teaching sites with regard to POCUS. The results from this study will help prioritize training needs for educators tasked with POCUS curriculum development and implementation.

## Methods

### Study design

This multi-center cross-sectional survey study was undertaken at five Canadian universities: the Universities of Calgary, Alberta, British Columbia, Ottawa and Western University. The respective Ethics Board at each of the five universities approved this study.

### Participants

All Internal Medicine residents (postgraduate year [PGY] 1–4), as well as General Internal Medicine subspecialty trainees (PGY 4–5) during the year 2015–2016 from the five institutions were invited to participate in this study (*n* = 598). Only those who consented to the survey were included in the study.

### Survey development

To support the content development of our survey, key texts and articles on POCUS were reviewed [31–35]. An initial survey was drafted with input from two researchers (KW and IM) in July 2015, containing a list of 15 diagnostic applications, 10 procedures, and 80 basic knowledge items. For each survey item on diagnostic applications and procedures, two questions were asked: 1) How applicable is the application/procedure to patient care in internal medicine? 2) What is the participant’s skill in that area? For knowledge items, only self-reported level of knowledge was asked.

This survey was then piloted with 8 non-internal medicine residents in order to obtain input on survey length, content, and clarity. Based on feedback from the pilot data, in particular with respect to the length of the initial survey, we substantially revised the survey. For diagnostic applications, items on A-lines and Z-lines were removed, as they were felt to be too specific. The addition of two diagnostic applications was suggested: deep vein thrombosis and hydronephrosis. For procedures, incision and drainage was removed as the skills involved were felt to be redundant with the skills involved in abscess aspiration. Lastly, many of the 80 items on POCUS knowledge [26] were felt to be too specific, resulting in a survey that was unacceptably long. Ultimately, knowledge items were grouped into broader categories. The final survey included 15 diagnostic applications, 9 procedures, and 18 knowledge items, in addition to questions on baseline demographic data (see Additional file 1).

Using an online survey tool (SurveyMonkey Inc. San Mateo, California, USA; www.surveymonkey.com), the final survey was distributed to the trainees between April and June 2016. Up to two reminder emails were sent between two and 8 weeks to maximize participant response rate. As this study was unfunded, no incentives were used in this study at any study site.

### Study outcomes

Perceived applicability of diagnostic applications and procedures to the practice of internal medicine was assessed using a 5-point Likert scale, where 1 = not at all applicable and 5 = very applicable. Self-reported skill level and knowledge was assessed using a 5-point Likert scale, where 1 = very poor and 5 = very good. We defined skill gap as the difference between perceived applicability and self-reported skill level.

### Statistical analysis

Data were analyzed using standard descriptive statistical techniques. Comparisons of continuous variables between groups were performed using Student’s *t*-tests. Categorical variables were compared with the use of Fisher’s exact tests and chi-square tests. All analyses were performed using SAS version 9.4 (SAS Institute Inc., Cary, NC, USA).

## Results

A total of 253 of 598 residents participated in our study for a cumulative response rate across all centers of 42%. We excluded data from 15 participants in a single center, as this center (Center E) did not achieve a response rate of > 40% (achieved only 12%). Further, baseline demographics of survey respondents from Center E differed significantly from respondents from Centers A to D in terms of procedural experience and in reported access to ultrasound preceptors (Table 1). The 4 centers included in this study had a mean response rate of 51 ± 10%, with a total of 238 participants included in our final analyses.

**Table 1:** Baseline demographics and experience of survey participants, presented as number (%).^a^

	All participants (*N* = 253)	Centers A to D (*N* = 238)	Center E (*N* = 15)	*p*-value
Post-graduate year (PGY)				
PGY-1	74 (29)	71 (30)	3 (20)	0.49
PGY-2	73 (29)	67 (28)	6 (40)	
PGY-3	65 (26)	62 (26)	3 (20)	
PGY-4	15 (6)	14 (6)	1 (7)	
PGY-5	16 (6)	14 (6)	2 (13)	
Gender				
Males	132 (52)	122 (51)	10 (67)	0.43
Females	109 (43)	104 (44)	5 (33)	
Number of ultrasound-guided paracenteses performed				
None	23 (9)	22 (9)	1 (7)	0.01
1–2	52 (21)	51 (21)	1 (7)	
3–5	73 (29)	72 (30)	1 (7)	
6–9	43 (17)	39 (16)	4 (27)	
10 or more	52 (21)	44 (18)	8 (53)	
Number of ultrasound-guided thoracenteses performed				
None	41 (16)	41 (17)	0	0.01
1–2	74 (29)	71 (30)	3 (20)	
3–5	66 (26)	63 (26)	3 (20)	
6–9	36 (14)	29 (12)	7 (47)	
10 or more	25 (10)	23 (10)	2 (13)	
Number of ultrasound-guided central line insertions performed				
None	35 (14)	34 (14)	1 (7)	0.17
1–2	27 (11)	26 (11)	1 (7)	
3–5	39 (15)	39 (16)	0	
6–9	42 (17)	37 (16)	5 (33)	
10 or more	99 (39)	91 (38)	8 (53)	
Number of ultrasound-guided peripheral intravenous catheterizations performed				
None	173 (68)	167 (70)	6 (40)	0.004
1–2	26 (10)	20 (8)	6 (40)	
3–5	26 (10)	24 (10)	2 (13)	
6–9	5 (2)	4 (2)	1 (7)	
10 or more	11 (4)	11 (5)	0	
How often learners wanted to perform an US-guided procedure but was not able to do so due to lack of supervisor/teacher (and not because of lack of equipment):^b^ Mean ± standard deviation	2.1 ± 0.8	2.1 ± 0.8	1.7 ± 0.6	0.04

^a^Not all participants responded to all questions

^b^Responses in Likert scale from 1 to 4, where 1 = never and 4 = most of the time

### Diagnostic applications

**Fig. 1:**
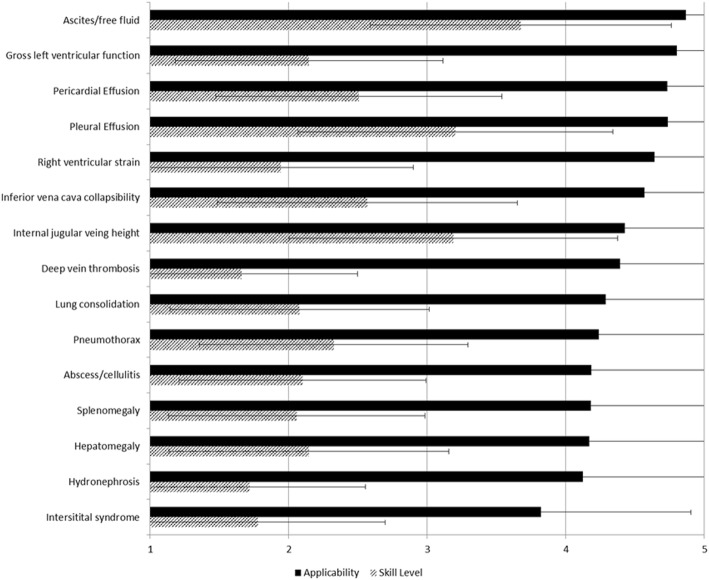
Diagnostic applications and their perceived applicability to the practice of internal medicine (rated on a 5-point Likert scale, where 1 = very not applicable and 5 = very applicable, black solid bars) and participants’ reported skill level (where 1 = very poor and 5 = very good, dashed bars)

Participants felt the following three diagnostic uses of ultrasound were most applicable to patient care in internal medicine: *identifying ascites/free fluid* [mean applicability score of 4.9 ± standard deviation (SD) 0.4]; *gross left ventricular function* (mean 4.8 ± SD 0.5) and *pericardial effusion* (mean 4.7 ± SD 0.5, Fig. 1). Participants reported lowest skill levels in *identifying deep vein thrombosis* (mean skill level 1.7 ± SD 0.8), *hydronephrosis* (mean 1.7 ± SD 0.8), and *pulmonary interstitial syndrome* (mean 1.8 ± SD 0.9). Skill gaps (difference between perceived applicability and self-reported skill level) were highest for *identifying deep vein thrombosis* (mean gap 2.7 ± SD 1.1), *right ventricular strain* (mean 2.7 ± SD 1.1), and *gross left ventricular function* (mean 2.7 ± SD 1.0).

### Procedures

**Fig. 2:**
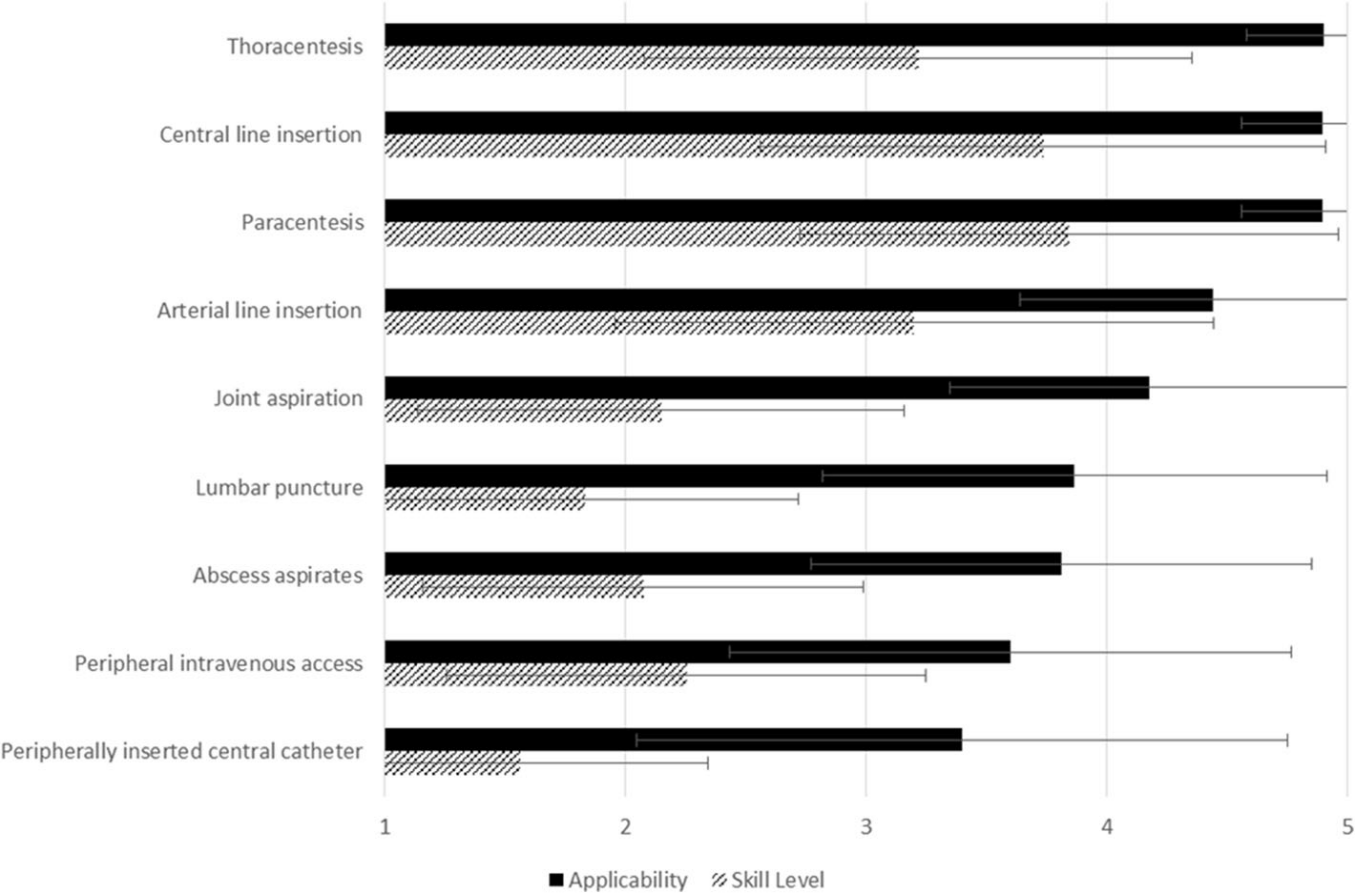
Bedside ultrasound-guided procedures and the perceived applicability to the practice of internal medicine (rated on a 5-point Likert scale, where 1 = very not applicable and 5 = very applicable, black solid bars) and participants’ reported skill level (where 1 = very poor and 5 = very good, dashed bars)

Residents identified the following ultrasound-guided procedures as most applicable to internal medicine: *thoracentesis* (mean applicability score 4.9 ± SD 0.3), *central line insertion* (mean 4.9 ± SD 0.3), and *paracentesis* (mean 4.9 ± SD 0.3, Fig. 2). Participants reported lowest skill levels in using ultrasound for: *peripherally inserted central catheter* (mean skill level 1.6 ± SD 0.8), *lumbar puncture* (mean 1.8 ± 3.9), and *superficial abscess aspiration* (mean 2.1 ± 0.9). Skill gaps were highest for *lumbar puncture* (mean gap 2.1 ± SD 1.4), *joint aspiration* (mean 2.0 ± 1.3), and *peripherally inserted central catheter* (mean 1.8 ± SD 1.3).

### Knowledge

**Fig. 3:**
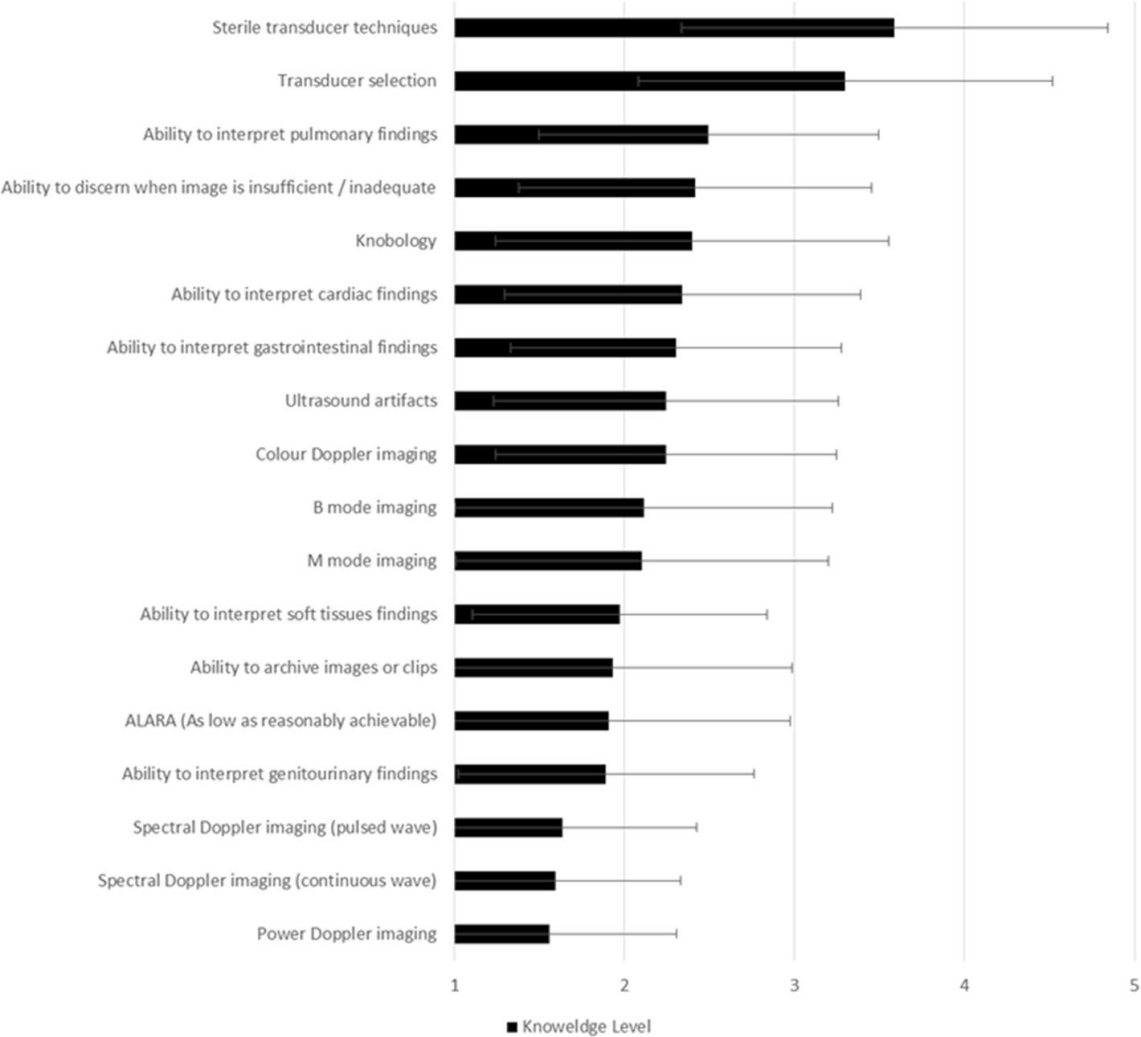
Self-report knowledge level in items related to point-of-care ultrasound knowledge (rated on a 5-point Likert scale, where 1 = very poor and 5 = very good)

Participants reported highest knowledge levels in: *sterile transducer techniques* (mean skill level 3.6 ± SD 1.3), *transducer selection* (mean 3.3 ± SD 1.2), and *ability to interpret pulmonary findings* (mean 2.5 ± SD 1.9, Fig. 3). Participants reported lowest knowledge levels in: *power Doppler imaging* (mean 1.6 ± SD 0.7), *continuous wave spectral Doppler imaging* (mean 1.6 ± SD 0.7), and *pulsed wave spectral Doppler imaging* (mean 1.6 ± SD 0.8).

## Discussion

This study demonstrates the perceived applicability to the practice of internal medicine, self-reported skill/knowledge level, and skill gap of 15 POCUS diagnostic applications, 9 ultrasound-guided procedures, and 18 POCUS knowledge items for Canadian internal medicine residents at four training sites. Our results suggest that learners felt using ultrasound to identify ascites/abdominal free fluid, gross left ventricular function and pericardial effusion were most applicable to the practice of internal medicine while least skillful in assessing for deep vein thrombosis and focused cardiac ultrasound assessments. POCUS was felt to be most applicable for the guidance for thoracentesis, central line insertion and paracentesis. Self-reported proficiency was lowest in peripherally inserted central catheter, lumbar puncture, and superficial abscess drainage. Lastly, learners reported overall low level of knowledge of POCUS, especially with respect to Doppler imaging.

Our results mirror those from the University of Illinois where learners reported low overall competence in POCUS [28] and the University of Toronto where only 21% of learners reported comfort in using POCUS for procedures [30]. Our present study adds to existing literature by providing additional granularity on gaps in specific ultrasound diagnostic skills, procedural skills and areas of knowledge. Time and costs of training are cited barriers to implementing a POCUS curriculum for internal medicine [29, 30, 36]. These results can help internal medicine educators who are tasked with implementing a POCUS curriculum focus their efforts on applications, procedures, or knowledge that are perceived to be the most applicable, where the learners report the least skills/knowledge, and/or where skill gaps are the highest, which takes into account both applicability and reported levels of skills and knowledge.

Our study has a few limitations. First, our results are based on learner self-report and the accuracy of self-assessed competence in skills and knowledge is questionable [37, 38]. Therefore, curriculum efforts should not necessarily ignore applications, procedures, or knowledge in which learners report high competence. Further, our definition of skill gap is only a surrogate measure, based on the difference between reported applicability and skills. Inaccurate assessment of either (or both) of these variables may render the interpretability of the skill gap measure questionable. Second, although this study provides the learner’s perspective on POCUS, curriculum implementation should take into account other factors, such as which POCUS applications can be mastered within constraints of each local curriculum setting (e.g. time requirement for various skill acquisition and availability of trained preceptors). Time requirements and trained preceptors are important considerations in curriculum design. We recommend that our results be triangulated with educators’ perspectives, which were sought based on clinical and educational needs and evidence, education feasibility, as well as consideration of patient safety issues [25]. These principles may not have been considered by the learners during survey response. Third, contrary to our expectations, our participants felt that the identification of interstitial syndrome [39] has low applicability to internal medicine. Existing data suggest that lung ultrasound is in fact highly applicable to internal medicine as it improves diagnostic accuracies and narrowing of differential diagnoses [40, 41]. In retrospect, it is likely that the term “interstitial syndrome,” while concordant with international recommendations [39], may be unfamiliar to our survey participants. The reported low skill level in identifying interstitial syndrome supports our hypothesis that our survey participants may not be familiar with the concept. As such, we would not recommend deferring the training of lung ultrasound to internal medicine residents based on our survey results. Fourth, our surveys were administered to Canadian internal medicine trainees at five training sites only and consequently, generalizability may be limited. Lastly, despite our best efforts, our overall response rate was only 42%, while not entirely out of keeping with physician survey response rate in general [42, 43], is lower than we would like.

Despite these limitations, to our knowledge, this study reports results from the largest multicenter internal medicine learners. As such, their collective experience and opinions regarding skill gaps are important to consider in our efforts to integrate POCUS safely into the practice of internal medicine [40].

## Conclusions

Our multi-center survey results from Canadian internal medicine residents suggest that learners found POCUS highly applicable to the practice of internal medicine, especially for identifying ascites and cardiac findings and for guiding central line insertion, paracentesis, and thoracentesis. Significant gaps were reported in skills and knowledge. Development of POCUS education should take these results into consideration when deciding where to focus curriculum efforts.

## Additional file

Additional file 1: Point-of-care ultrasound needs assessment survey administered. (PDF 324 kb)

## Abbreviations

PGY: Postgraduate year
POCUS: Point-of-care ultrasound
SD: Standard deviation
