# Supplementary material for: Point of care ultrasound training for internal medicine: a Canadian multi-centre learner needs assessment study
Source: BMC Med Educ. 2018 Sep 20;18:217. doi: 10.1186/s12909-018-1326-8 (PMC6149066; doi:10.1186/s12909-018-1326-8)
Supplement: Supplementary file 1 — Point-of-care ultrasound needs assessment survey administered. (PDF 324 kb) [file 12909_2018_1326_MOESM1_ESM.pdf]

Demographic

1. What year of residency are you in?

- ◯ PGY-1
- ◯ PGY-2
- ◯ PGY-3
- ◯ PGY-4
- ◯ PGY-5
- ◯ PGY-6 and beyond

2. Gender

- ◯ Male
- ◯ Female

3. How many procedures have you performed to date **with ultrasound**?

	0	1-2	3-5	6-9	10 or more
Abdominal paracentesis	◯	◯	◯	◯	◯
Thoracentesis	◯	◯	◯	◯	◯
Central line insertion	◯	◯	◯	◯	◯
Peripheral IV insertion	◯	◯	◯	◯	◯

4. How many times have you encountered a situation when you would have wanted to perform an US-guided procedure but was not able to do so because of the lack of **supervisor/teacher** (and not because of the lack of US machine)?

- ◯ Never
- ◯ A few times
- ◯ Many times
- ◯ Most of the time
- ◯ Not applicable (please specify)

Interest in learning

5. How applicable to your patient care and what are your personal needs in learning the following ultrasound applications?

	How applicable to patient care in Internal Medicine	What are your own current skills/knowledge
Determining the height of the internal jugular vein		
Measuring IVC diameter / collapsibility index		
Identifying pleural effusion		
Identifying pneumothorax		
Identifying interstitial syndrome		
Identifying lung consolidation		
Identifying hepatomegaly		
Identifying splenomegaly		
Identifying ascites/free fluid		
Identifying abscess/cellulitis		
Identifying DVT		
Identifying hydronephrosis		
Identifying gross LV function		
Identifying RV strain		
Identifying pericardial effusion		
Using US for central line insertion		
Using US for arterial line insertion		
Using US for peripheral IV insertion		
Using US for thoracentesis		

	How applicable to patient care in Internal Medicine	What are your own current skills/knowledge
Using US for paracentesis		
Using US for joint aspiration		
Using US for lumbar puncture		
Using US for superficial abscess aspirates for sampling		
Using US for PICC line insertion		

Other (please specify)

Basic Ultrasound Knowledge and Use

The following section seeks your general knowledge and ultrasound (US) skills. How would you rate your own knowledge/skills on the following?

6. How would you rate your current level of knowledge or skills in the following domains?

	Very Poor	Poor	Fair	Good	Very Good
The ALARA principle (As low as reasonably achievable)	○	○	○	○	○
B mode imaging	○	○	○	○	○
M mode imaging	○	○	○	○	○
Colour Doppler imaging	○	○	○	○	○
Spectral Doppler imaging - Pulsed wave	○	○	○	○	○
Spectral Doppler imaging - continuous wave	○	○	○	○	○
Power Doppler imaging	○	○	○	○	○
Ultrasound artifacts	○	○	○	○	○
Transducer selection	○	○	○	○	○
Sterile transducer techniques	○	○	○	○	○
Knobology	○	○	○	○	○
Ability to interpret findings - cardiac system	○	○	○	○	○
Ability to interpret findings - pulmonary system	○	○	○	○	○
Ability to interpret findings - GI system	○	○	○	○	○
Ability to interpret findings - GU system	○	○	○	○	○
Ability to interpret findings - soft tissues	○	○	○	○	○
Ability to discern when image is insufficient / inadequate	○	○	○	○	○
Ability to archive images or cine-clips	○	○	○	○	○

7. Do you have any other comments or concerns?
